# Fasting apolipoprotein B48 is associated with large artery atherosclerotic stroke: a case-control study

**DOI:** 10.1038/s41598-019-40385-0

**Published:** 2019-03-06

**Authors:** Jing Tian, Hong Chen, Ping Liu, Chun Wang, Yong Chen

**Affiliations:** Department of neurology, People’s Hospital of Deyang City, Taishian North Road 173, Deyang City, 618000 China

## Abstract

Fasting Apolipoprotein B48 (ApoB48) is reported to be a well surrogate marker for postprandial lipidemia and have been repeatedly associated with cardiovascular disease. However, whether ApoB48 is also a risk factor for ischemic stroke have not been reported. In this study, our object is to explore the relationship between fasting plasma ApoB48 levels and the large artery atherosclerotic (LAA) stroke.A 1:1 age-(±2), gender-matched case-control study was conducted. LAA patients and healthy controls admitted to our center were prospectively recruited. Clinical data were collected and enzyme-linked immunosorbent assay (ELISA) was used to measure the fasting plasma ApoB48 levels.A cohort of 234 LAA stroke patients and 234 controls were enrolled. Fasting plasma ApoB48 levels were significantly higher in LAA stroke patients than in controls (4.76(3.46) vs 4.00(2.4), *P* < 0.001). Conditional multivariable analyses indicated that fasting ApoB48 levels were associated with LAA stroke (odds ratio: 1.18; 95% confidence interval: 1.04–1.35; P = 0.014).Our study indicates that increased fasting plasma ApoB48 may be a risk factor for LAA stroke.

## Introduction

Hypertriglyceridemia (HTG) is a common type of dyslipidemia, especially in China. HTG could initiate and promote the atherosclerotic process, which could in turn accelerate cardiovascular disease^1^. Therefore, HTG is thought to be a risk factor for ischemic stroke. A lot of clinical studies have been conducted to uncover the relationship between HTG and ischemic stroke. However, the results are controversial and no consensus has been made until now. Most of the studies using fasting triglyceride levels as a marker for ischemic stroke have been conflicting. Several studies have revealed that elevated fasting triglyceride level is a risk factor for ischemic stroke^2,3^. However, these results cannot be verified in the ARIC study^4^ and the Physicians’ Health Study^5^, in which fasting TG levels had no relationship with ischemic stroke. In contrast to the above inconsistence findings, nonfasting triglyceride levels were reported consistently to be a risk factor for ischemic stroke in the Copenhagen City Heart Study^6,7^ and Women’s Health Study^8^. These results indicated that nonfasting or postprandial triglyceride levels may be a more suitable biomarker for ischemic stroke and atherosclerotic disease compared to fasting triglyceride levels. However, multiple factors are associated with elevated nonfasting triglyceride levels, and no standardization or consensus has been made so far for the measurement of nonfasting triglyceride levels^1^. Therefore, scientists are trying to find a more stable surrogate biomarker.

Nonfasting hypertriglyceridemia is due to the accumulation of triglyceride-rich chylomicrons and their partially hydrolyzed chylomicrons, which are highly atherogenic. The absorptive cells in the small intestine produce Apolipoprotein B48 (ApoB48) to form the chylomicrons. Each chylomicron particle contains one molecule of ApoB48^9^. In addition, previous studies have indicated that the fasting concentration of ApoB48 can be a good surrogate marker for the degree of postprandial lipidemia^10,11^. Therefore, the fasting level of ApoB48 may be a good biomarker for atherosclerotic diseases including cardiovascular disease and ischemic stroke. Clinical studies have proved that the fasting level of ApoB48 is associated with carotid atherosclerosis^12–16^, peripheral artery disease^17^ and coronary artery disease^18,19^. However, the association between fasting ApoB48 and ischemic stroke is still unknown. Thus, our objective in this study was to explore the relationship between fasting plasma ApoB48 levels and the large artery atherosclerotic (LAA) stroke.

## Results

**Figure 1:**
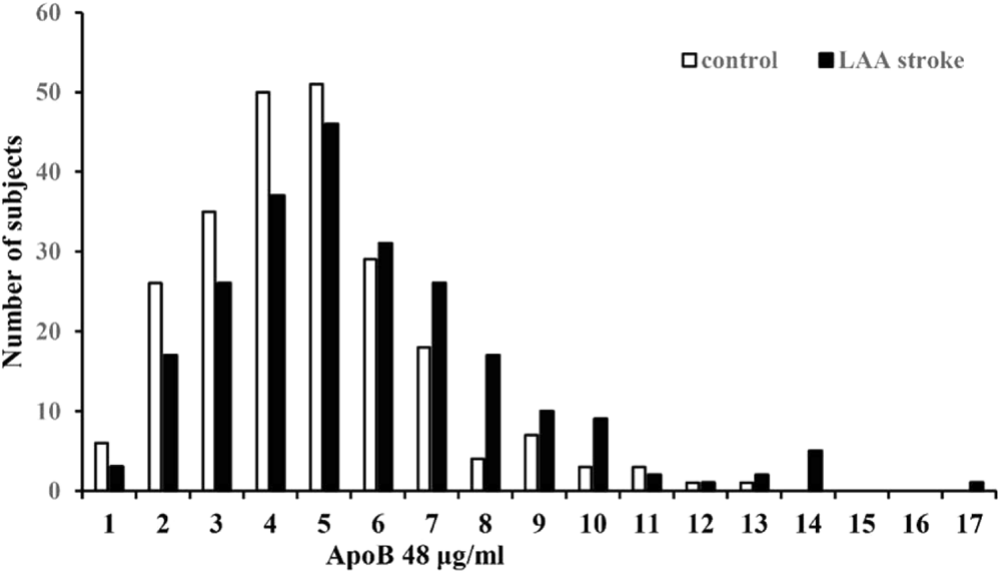
Distribution of fasting plasma ApoB48 Levels in controls and Patients with LAA stroke. Plasma concentrations of ApoB48 = 1 represents concentrations between 0 and 1.0 μg/mL.

During the study period, 234 patients meeting inclusion criteria were included in this study. There were 147 (62.82%) males and the mean age was 59.97 ± 11.45 years. Controls were recruited with a 1:1 age-(±2), and gender-matched fashion during the same study period. The mean age of controls was 59.76 ± 11.39 years. Table 1 summarizes the demographic and clinical features of the controls and LAA stroke patients. Compared with the controls, the patients with LAA stroke had higher prevalence of vascular risk factors (hypertension, diabetes, smoking, drinking) and higher mean values of BMI, and TG levels (all p < 0.05). However, the control participants had higher plsama levels of HDL-C than the LAA stroke patients (p = 0.008). Fasting plasma ApoB48 levels ranged from 0 to 13 μg/mL in control participants, and from 0 to 18 μg/mL in LAA stroke patients (Fig. 1). The distribution of ApoB48 levels in controls and LAA stroke patients was different. Further analysis revealed that the plasma levels of ApoB48 were higher in the patients with LAA stroke than in the controls (4.76(3.46) vs 4.00(2.4), P < 0.001). When the plasma levels of ApoB48 were dichotomized at 5.29 μg/mL according to the ROC curve analysis, the patients in LAA stroke group had a significantly higher proportion of high ApoB48 levels (42.74% vs. 23.50%, p < 0.001), compared with controls.

**Table 1:** Clinical characteristics of the controls and patients with LAA stroke.

variables	Control (n = 234)	LAA (n = 234)	*P* value
Male, n (%)	147 (62.82%)	147 (62.82%)	1
Age, y	59.76 ± 11.39	59.97 ± 11.45	0.849
BMI	23.55 ± 3.39	24.31 ± 3.08	0.012
Hypertension, n (%)	45 (19.23%)	167 (71.37%)	<0.001
Smoking, n (%)	67 (28.63%)	101 (43.16%)	<0.001
Diabetes mellitus, n (%)	23 (9.83%)	83 (35.47%)	<0.001
Drinking	42 (17.95%)	61 (26.07%)	0.044
Fasting glucose, mmol/L	5.44 (1.06)	5.79 (2.99)	0.045
Creatinine, μmol/L	72.4 (17.15)	73.05 (26.83)	0.449
TC, mmol/L	4.6 (1.14)	4.61 (1.53)	0.806
TG, mmol/L	1.45 (1.05)	1.74 (0.96)	<0.001
HDL, mmol/L	1.31 (0.58)	1.26 (0.36)	0.008
LDL, mmol/L	2.74 (0.97)	2.75 (1.20)	0.372
ApoB48 μg/mL	4.00 (2.40)	4.76 (3.46)	<0.001
ApoB48 > 5.29 μg/mL, n (%)	55 (23.50%)	100 (42.74%)	<0.001

Data are expressed as mean ± standard deviation or median (interquartile range) for numerical variables and counts (%) for categorical variables. Statistically significant differences were determined using the χ2 test for categorical variables, and Student t test for continuous variables, between different groups. TC indicates total cholesterol; TG, triglycerides; HDL, high-density lipoprotein; LDL, low-density lipoprotein; LAA, large-artery atherosclerosis stroke; BMI, body mass index.

**Figure 2:**
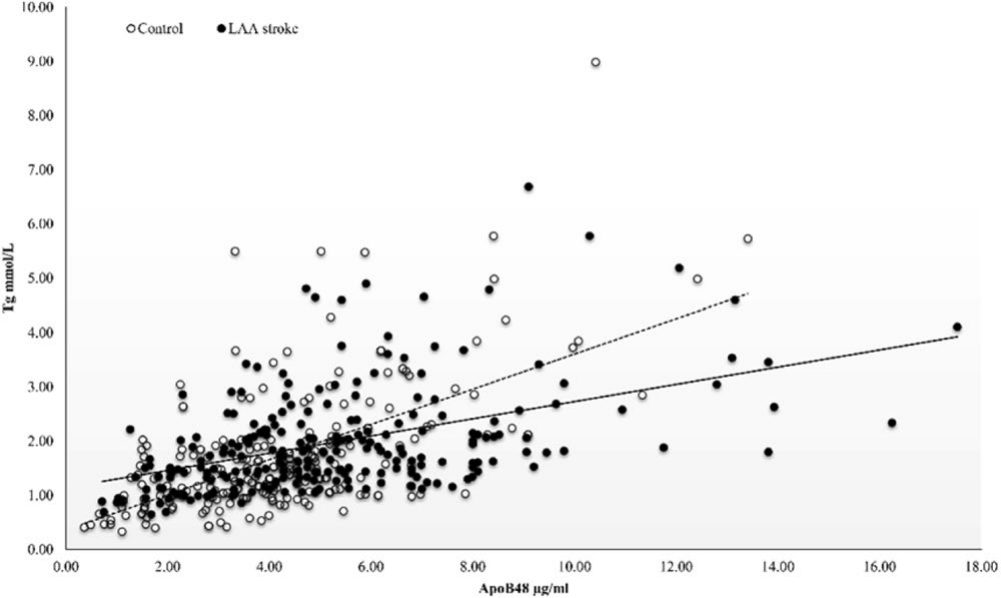
Correlation between fasting plasma ApoB48 Levels and TG levels. Pearson correlation analysis revealed that there was a linear relationship between ApoB48 levels and TG levels in controls (p < 0.001, r = 0.584) and in LAA stroke patients (p < 0.001, r = 0.464).

**Figure 3:**
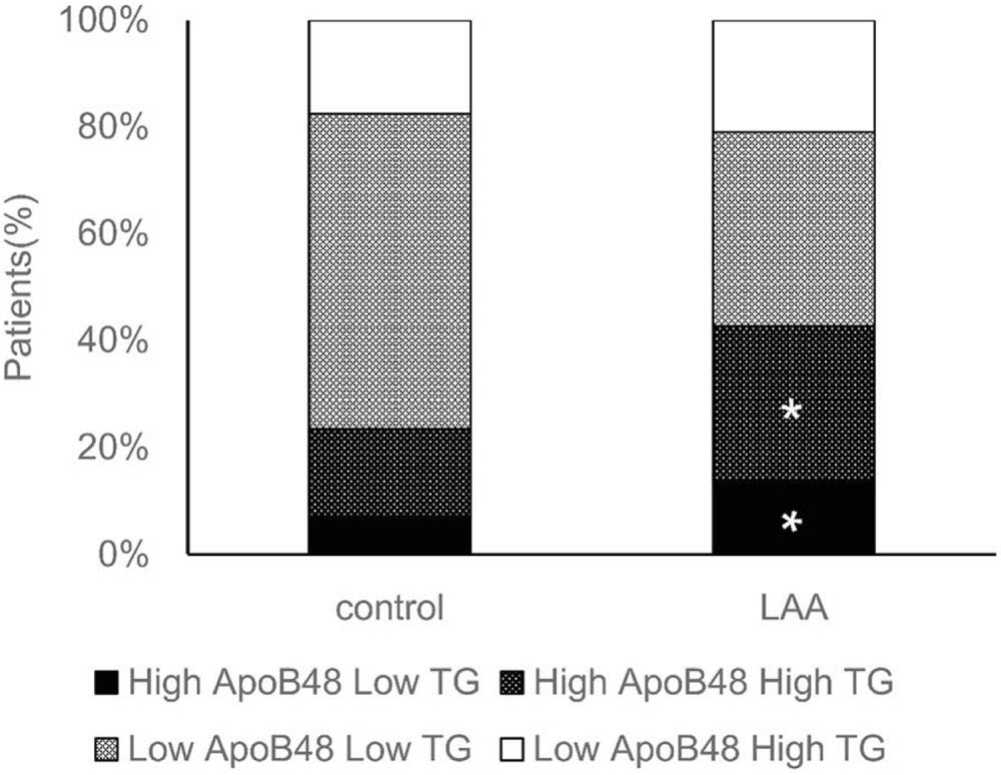
Plasma levels of ApoB48 and TG in LAA stroke patients and controls. Participants were divided into four groups according to their fasting levles of ApoB48 (cutoff value, 5.29 μg/ml) and TG (cutoff value, 1.74 mmol/L). A high ApoB48 level was more common in LAA stroke patients than in controls even if the TG levels were low. *p < 0.01 vs. Control group.

Further analysis indicated that there was a linear relationship between ApoB48 levels and TG levels in controls (p < 0.001, r = 0.584) and in LAA stroke group (p < 0.001, r = 0.464) (Fig. 2). TG level were dichotomized at 1.74 mmol/L according to the ROC curve analysis and patients were separated into four groups by combining ApoB48 and TG level: High ApoB48 Low TG, High ApoB48 High TG, Low ApoB48 Low TG, and Low ApoB48 High TG group. The high levels of both ApoB48 and TG was more common in LAA stroke patients than in controls (Figs. 3, 29.06% vs 16.67%, p < 0.001). Furthermore, the proportion of participants with High ApoB48 Low TG in LAA stroke group (13.67%) was significantly greater than in controls (6.84%).

The correlation of ApoB48 with LAA stroke could be confounded by various factors. Therefore, further analyses were conducted to uncover whether ApoB48 is independently associated with LAA stroke. Those variables with p < 0.10 in the univariable analyses were included into further analysis model. After adjusting for hypertension, diabetes mellitus, BMI, smoking, drinking, TG, and HDL, fasting plasma ApoB48 levels were significantly associated with LAA stroke (OR = 1.18, 95% CI = 1.04–1.35, P = 0.014) (Table 2). However, the TG levels were not associated with LAA stroke regardless of inclusion of ApoB48 in the model. When the dichotomized variable “ApoB48 > 5.29 μg/mL” was entered into the model (Supplementary Table 1), a high ApoB48 level was also significantly associated with LAA stroke (OR = 3.50, 95% CI = 1.69–7.26, P = 0.001).

**Table 2:** Univariate and multivariate conditional logistic regression analysis for risk Factors in patients with LAA stroke.

Variables	Univariate			Multivariate		
	OR	95%CI	*P* value	OR	95%CI	*P* value
Hypertension	9.13	5.36–15.56	<0.001	8.04	4.43–14.59	<0.001
Smoking	2.26	1.44–3.55	<0.001	2.42	1.22–4.80	0.012
Diabetes mellitus	4.53	2.68–7.66	<0.001	2.67	1.29–5.53	0.008
Drinking	1.68	1.05–2.68	0.03	1.57	0.73–3.36	0.248
BMI	1.08	1.02–1.15	0.01	1.07	0.98–1.17	0.15
TG	1.19	1.00–1.40	0.04	0.86	0.64–1.16	0.31
HDL	0.54	0.33–0.87	0.01	1.05	0.53–2.08	0.89
ApoB48	1.20	1.10–1.30	<0.001	1.18	1.04–1.35	0.014

Hypertension, Smoking, Drinking, Diabetes mellitus, BMI, TG, HDL, and ApoB48 were entered into the multivariate conditional logistic regression model. Similar results were obtained if forward stepwise or backward stepwise model was used and if fasting glucose replaced Diabetes mellitus in the models.

## Discussion

In this study, we demonstrated that, for the first time, fasting ApoB48 levels were higher in LAA stroke patients, and a higher ApoB48 level (ApoB48 > 5.29 μg/mL) was nearly two times more prevalent in LAA stroke patients (42.74%) than in controls (23.50%). In multivariable analysis, fasting ApoB48 levels (OR = 1.18, 95% CI = 1.04–1.35, P = 0.014) and a high ApoB48 level (OR = 3.50, 95% CI = 1.69–7.26, P = 0.001) were associated with LAA stroke independent of conventional risk factors. Fasting TG levels were positively correlated with ApoB48 levels; however, the fasting plasma levels of TG were not an independently risk factor for LAA stroke.

Postprandial HTG is one of the independent risk factors for atherosclerosis. Moreover, previous large cohort studies have proved that postprandial TG levels may be a more suitable marker for ischemic stroke than fasting TG level. However, there is no standardized way to measure postprandial TG levels. Postprandial TG levels are traditionally evaluated as the response to a standard rich, high-fat meal. This test needs 6 to 8 hours and requires the patients’ cooperation. Therefore, the discovery of surrogate markers of postprandial HTG, which could be easily measured in routine clinical practice, will be useful to evaluate the stroke risk. As one chylomicron particle contains one molecule of ApoB48, the plasma level of ApoB48 indicates the number of postprandial particles [9].Fasting levels of ApoB48 closely correlate with postprandial TG levels after a lipid meal [10]. This information suggests that fasting ApoB48 level is a surrogate markers of postprandial HTG. Previous studies have shown that fasting ApoB48 could be a risk factor for atherosclerosis and atherosclerotic cardiovascular diseases. The present cross-sectional study, which shows that fasting ApoB48 is associated with LAA stroke, adds new information to this connection.

Our findings that fasting ApoB48 is associated with LAA stroke indicates that ApoB48-containing lipoproteins may take part in the development of atherosclerosis^20^. A lot of experimental studies have indicated the possible underlying mechanisms. ApoB48 has a binding site to arterial wall proteoglycans^21^. As a result, ApoB48 was found in human atherosclerotic plaques from femoral and carotid endarterectomy samples^22^. *In vitro* studies have indicated that chylomicron remnants could be uptaken by mouse peritoneal macrophages and human monocyte-derived macrophages though multiple mechanisms^23^. Moreover, chylomicron remnants could induce monocyte chemoattractant protein-1 expression via p38 MAPK activation and regulate early growth response factor-1 in vascular smooth muscle cells^24,25^. In addition, chylomicron remnants could increase the production of plasminogen activator inhibitor-1 (PAI-1) and enhance apoptosis in endothelial cells^26^. These studies provide the physiopathological mechanisms to support our findings.

There are some limitations in this study. First, as a cross-sectional study, this study cannot help to make causal inferences. Second, the fasting levels of ApoB48 may change after disease onset. However, we recruited patients who were admitted in our hospital within 24 hours after disease onset, and existing study show that lipid concentrations have not significant changes during the first days after stroke onset^27^.

In conclusion, fasting plasma ApoB48 levels were significantly correlated with the prevalence of LAA stroke. Therefore, ApoB48 may be a new marker for LAA stroke, as well as a possible therapeutic target.

## Material and Methods

### Patients and controls

This study was approved by the Ethics Committees of the People’s Hospital of Deyang City. Informed consent have been obtained from every participant.All methods were performed in accordance with the relevant guidelines and regulations. From February 2015 to December 2017, consecutive ischemic patients who were admitted to our hospital were prospectively screened for enrollment. The diagnosis of LAA stroke was confirmed by two neurologists. The inclusion criteria was admission within 24 hours after onset of ischemic stroke. The exclusion criteria were as follows: (1) having a previous history of stroke or ischemic heart disease; (2) having received treatment before admission including statin treatment; (3) not having fasting plasma drawn within 24 hours after admission; (4) having incomplete data for stroke etiology and/or two or more stroke etiology; (5) having systematic diseases. We recruited healthy volunteers as controls who received health examinations in our hospital during the same study period. Those volunteers who were free of history of stroke, myocardial infarction, and systematic diseases were included in this study. Ultimately, 234 LAA stroke patients and 234 healthy volunteers were recruited in this study.

### Assessment of stroke risk factors

The demographic characteristics, past medical history and clinical data sof the controls and patients were recorded prospectively. The common vascular risk factors including hypertension, diabetes mellitus, drinking, smoking and heart disease were recorded. Diabetes mellitus and hypertension were defined according with the diagnosis guidelines. Smoking was defined as smoking equal to or more than one cigarette per day for one year or more. Alcohol consumption was defined as a past or current history of drinking more than once per day for more than 1 year. Heart disease was defined as if a subject had one or more heart disease, such as myocardial infarction, and atrial fibrillation. Magnetic resonance imaging (MRI) with diffusion weighted imaging, MR or CT angiography, carotid duplex ultrasonography, transthoracic echocardiography, 24-h Holter monitoring, and other routine admission laboratory tests were conducted to help to assess the stroke subtype. Transesophageal echocardiography was also performed if needed. LAA ischemic stroke was assessed by two independent neurologists according to the Trial of Org 10172 in the Acute Stroke Treatment study^28^.

All LAA stroke patients and controls had fasting lipid panels drawn after an overnight fast. Total cholesterol, triglycerides, HDL, LDL were measured by standard laboratory methods on fresh plasma. In addition, additional plasma of every patient and control were frozen in a −80 °C freezer for later use.

### Enzyme-Linked Immunosorbent Assay

Plasma fasting ApoB48 levels were quantified by enzyme-linked immunosorbent assay (ELISA) according to the manufacturer’s instruction (Fujirebio, Tokyo, Japan)^29^. The concentrations of ApoB48 were measured in batches. Reference plasma samples were pooled from 20 healthy controls and added to each plate to minimize plate-to-plate variations. Board-certified laboratory technicians performed the measurement and they were blinded to group information. The reproducibility of the results was assessed via calculating the average coefficient of variation (CV) within plates and between plates. The mean intra-assay CV was <7%, and the interassay CV was <15%.

### Statistical analysis

Data were given as percentages, means and standard deviations or median and interquartile range, as appropriate. The continuous variables between groups were compared by Student’s t-test. The proportions variables between groups were compared by χ^2^ test. Multivariate analysis was conducted by conditional logistic regression to determine if the fasting ApoB48 level was independently related with LAA stroke after adjustment for common risk factors. The differences between groups were considered significant if the p-value was less than 0.05 (two tailed). Statistical Package for Social Science (SPSS, version 22) was used to conduct the statistical analyses.

The datasets generated during and/or analyzed during the current study are available from the corresponding author on reasonable request.

## Supplementary information

supplementary file

## References

[1] Kolovou GD (2011). Assessment and clinical relevance of non-fasting and postprandial triglycerides: an expert panel statement. Curr Vasc Pharmacol.

[2] Patel A (2004). Serum triglycerides as a risk factor for cardiovascular diseases in the Asia-Pacific region. Circulation.

[3] Berger JS (2012). Lipid and lipoprotein biomarkers and the risk of ischemic stroke in postmenopausal women. Stroke.

[4] Shahar E (2003). Plasma lipid profile and incident ischemic stroke: the Atherosclerosis Risk in Communities (ARIC) study. Stroke.

[5] Bowman TS (2003). Cholesterol and the risk of ischemic stroke. Stroke.

[6] Freiberg JJ, Tybjaerg-Hansen A, Jensen JS, Nordestgaard BG (2008). Nonfasting triglycerides and risk of ischemic stroke in the general population. JAMA.

[7] Varbo A (2011). Nonfasting triglycerides, cholesterol, and ischemic stroke in the general population. Ann Neurol.

[8] Bansal S (2007). Fasting compared with nonfasting triglycerides and risk of cardiovascular events in women. JAMA.

[9] Phillips ML (1997). A single copy of apolipoprotein B-48 is present on the human chylomicron remnant. J Lipid Res.

[10] Smith D, Watts GF, Dane-Stewart C, Mamo JC (1999). Post-prandial chylomicron response may be predicted by a single measurement of plasma apolipoprotein B48 in the fasting state. Eur J Clin Invest.

[11] Masuda D (2011). Fasting serum apolipoprotein B-48 can be a marker of postprandial hyperlipidemia. J Atheroscler Thromb.

[12] Meyer E (1996). Abnormal postprandial apolipoprotein B-48 and triglyceride responses in normolipidemic women with greater than 70% stenotic coronary artery disease: a case-control study. Atherosclerosis.

[13] Tanimura K (2008). Association of serum apolipoprotein B48 level with the presence of carotid plaque in type 2 diabetes mellitus. Diabetes Res Clin Pract.

[14] Nakatani K (2011). Serum apolipoprotein B-48 levels are correlated with carotid intima-media thickness in subjects with normal serum triglyceride levels. Atherosclerosis.

[15] Lapice E (2012). Fasting apolipoprotein B48 is associated with asymptomatic peripheral arterial disease in type 2 diabetic subjects: a case-control study. Atherosclerosis.

[16] Alipour A (2012). Exploring the value of apoB48 as a marker for atherosclerosis in clinical practice. Eur J Clin Invest.

[17] Mancera-Romero J (2013). Fasting apolipoprotein B48 is a marker for peripheral arterial disease in type 2 diabetes. Acta Diabetol.

[18] Masuda D (2012). Correlation of fasting serum apolipoprotein B-48 with coronary artery disease prevalence. Eur J Clin Invest.

[19] Mori K (2013). Fasting serum concentration of apolipoprotein B48 represents residual risks in patients with new-onset and chronic coronary artery disease. Clinica chimica acta; international journal of clinical chemistry.

[20] Masuda D, Yamashita S (2017). Postprandial Hyperlipidemia and Remnant Lipoproteins. J Atheroscler Thromb.

[21] Flood C (2002). Identification of the proteoglycan binding site in apolipoprotein B48. J Biol Chem.

[22] Pal S, Semorine K, Watts GF, Mamo J (2003). Identification of lipoproteins of intestinal origin in human atherosclerotic plaque. Clin Chem Lab Med.

[23] Fujioka Y, Cooper AD, Fong LG (1998). Multiple processes are involved in the uptake of chylomicron remnants by mouse peritoneal macrophages. J Lipid Res.

[24] Domoto K (2003). Chylomicron remnants induce monocyte chemoattractant protein-1 expression via p38 MAPK activation in vascular smooth muscle cells. Atherosclerosis.

[25] Takahashi Y (2005). Chylomicron remnants regulate early growth response factor-1 in vascular smooth muscle cells. Life Sci.

[26] Kawasaki S (2000). Chylomicron remnant induces apoptosis in vascular endothelial cells. Ann N Y Acad Sci.

[27] Weir CJ, Sattar N, Walters MR, Lees KR (2003). Low triglyceride, not low cholesterol concentration, independently predicts poor outcome following acute stroke. Cerebrovasc Dis.

[28] Adams HP (1993). Classification of subtype of acute ischemic stroke. Definitions for use in a multicenter clinical trial. TOAST. Trial of Org 10172 in Acute Stroke Treatment. Stroke.

[29] Sakai N (2003). Measurement of fasting serum apoB-48 levels in normolipidemic and hyperlipidemic subjects by ELISA. J Lipid Res.

