# Supplementary material for: Fasting apolipoprotein B48 is associated with large artery atherosclerotic stroke: a case-control study
Source: Sci Rep. 2019 Mar 6;9:3729. doi: 10.1038/s41598-019-40385-0 (PMC6403240; doi:10.1038/s41598-019-40385-0)
Supplement: Supplementary file 1 — supplementary file [file 41598_2019_40385_MOESM1_ESM.docx]

**Supplementary table one**: Univariate and Multivariate Conditional Regression Analysis for Risk Factors in patients with LAA stroke.

| Variables | Univariate | | | Multivariate | | |
| --- | --- | --- | --- | --- | --- | --- |
|  | OR | 95%CI | Pvalue | OR | 95%CI | P value |
| Hypertension | 9.13 | 5.36-15.56 | <0.001 | 8.92 | 44.77-16.69 | <0.001 |
| Smoking | 2.26 | 1.44-3.55 | <0.001 | 2.72 | 1.32-5.58 | 0.007 |
| Diabetes mellitus | 4.53 | 2.68-7.66 | <0.001 | 2.69 | 1.28-5.64 | 0.009 |
| Drinking | 1.68 | 1.05-2.68 | 0.03 | 1.45 | 0.66-3.21 | 0.356 |
| BMI | 1.08 | 1.02-1.15 | 0.01 | 1.08 | 0.99-1.19 | 0.082 |
| TG | 1.19 | 1.00-1.40 | 0.04 | 0.81 | 0.60-1.10 | 0.18 |
| HDL | 0.54 | 0.33-0.87 | 0.01 | 1.04 | 0.52-2.01 | 0.91 |
| ApoB48>5.29μg /mL | 2.45 | 1.62-3.72 | <0.001 | 3.50 | 1.69-7.26 | 0.001 |

Hypertension, Smoking, Drinking, Diabetes mellitus, BMI, TG, HDL, and ApoB48 (as a discontinue variable) were entered into the model. Similar results were obtained if fasting glucose replaced Diabetes mellitus in the model
